# Painful Menstruation

**Published:** 1899-03

**Authors:** J. E. Huffman

**Affiliations:** Healdsburg, Cal.


### PAINFUL MENSTRUATION.

J. E. Huffman, Healdsburg, Cal.

Miss M., Ogden, Utah, æt. 15; slender, blond, mild disposition. Sent her sister on July 7th, 1897, for medicine. Had the following symptoms: Menstrual flow began to-day; tosses about bed; cries and screams; is always kept in bed for about a week, though has had homœopathic (?) treatment for a week preceding each period, for some time. ℞ Pulsatilla, 2c; a powder in water to take three doses.

Heard no more of the case for some days, when I learned she was relieved in a few minutes, and went up the cañon two days later.

Next period much less pain, though she got her feet wet just before it came.

